# Biotransformation of artemisinin to a novel derivative via ring rearrangement by A*spergillus niger*

**DOI:** 10.1007/s00253-022-11888-0

**Published:** 2022-03-31

**Authors:** Jiaer Luo, Rebecca Mobley, Sian Woodfine, Falko Drijfhout, Paul Horrocks, Xiao-Dong Ren, Wen-Wu Li

**Affiliations:** 1 Department of Biopharmacy, School of Life Science, Jilin University, Changchun, 130012 China; 2 School of Medicine, Keele University, Staffordshire, ST5 5BG UK; 3 Chemical Sciences Research Centre, Keele University, Staffordshire, ST5 5BG UK; 4 School of Pharmacy and Bioengineering, Keele University, Stoke-on-Trent, ST4 7QB UK

**Keywords:** Biotransformation, Artemisinin, Kinetics, *Aspergillus niger*, Anti-plasmodial activity

## Abstract

**Abstract:**

Artemisinin is a component part of current frontline medicines for the treatment of malaria. The aim of this study is to make analogues of artemisinin using microbial transformation and evaluate their in vitro antimalarial activity. A panel of microorganisms were screened for biotransformation of artemisinin (**1**). The biotransformation products were extracted, purified and isolated using silica gel column chromatography and semi-preparative HPLC. Spectroscopic methods including LC-HRMS, GC–MS, FT-IR, 1D and 2D NMR were used to elucidate the structure of the artemisinin metabolites.^1^H NMR spectroscopy was further used to study the time-course biotransformation. The antiplasmodial activity (IC_50_) of the biotransformation products of **1** against intraerythrocytic cultures of *Plasmodium falciparum* were determined using bioluminescence assays. A filamentous fungus *Aspergillus niger* CICC 2487 was found to possess the best efficiency to convert artemisinin (**1**) to a novel derivative, 4-methoxy-9,10-dimethyloctahydrofuro-(3,2-i)-isochromen-11(4H)-one (**2**) via ring rearrangement and further degradation, along with three known derivatives, compound (**3**), deoxyartemisinin (**4**) and 3-hydroxy-deoxyartemisinin (**5**). Kinetic study of the biotransformation of artemisinin indicated the formation of artemisinin G as a key intermediate which could be hydrolyzed and methylated to form the new compound **2**. Our study shows that the anti-plasmodial potency of compounds **2**, **3, 4** and **5** were ablated compared to **1,** which attributed to the loss of the unique peroxide bridge in artemisinin (**1)**. This is the first report of microbial degradation and ring rearrangement of artemisinin with subsequent hydrolysis and methoxylation by *A.niger.*

**Key points:**

• *Aspergillus niger CICC 2487 was found to be efficient for biotransformation of artemisinin*

• *A novel and unusual artemisinin derivative was isolated and elucidated*

• The *peroxide bridge in artemisinin is crucial for its high antimalarial potency*

• *The pathway of biotransformation involves the formation of artemisinin G as a key intermediate*

**Supplementary Information:**

The online version contains supplementary material available at 10.1007/s00253-022-11888-0.

## Introduction

Artemisinin (**1**), qinghaosu, is a sesquiterpene lactone with an unique endoperoxide moiety first isolated from a Chinese medicinal plant in 1970s and showed extreme potency to malaria (Tu 2016). Artemisinin combination therapies are front-line medicines for the treatment of malaria. Artemisinin has also been intensively investigated for the treatment of cancer (Efferth 2017). Recent reports show the development and global spread of artemisinin resistance (Ashley et al. 2014; Balikagala et al. 2021; Miotto et al. 2020). This threat highlights the need to continue to search for novel antimalarial therapies, for example, novel analogues of artemisinin.

Many derivatives of artemisinin have been made chemically or biologically and tested for antimalarial (Kumari et al. 2019; Patel et al. 2021) and anticancer activity (Crespo-Ortiz and Wei 2012; Zhang et al. 2016). In particular, various artemisinin analogues including dihydroartemisinin, arteether, artemether, artesunate, artelinic acid, sodium artelinate and artesunic acid have been developed through chemical modification and approved for use (Kumari et al. 2019). Microbial transformation is the structural modification of complex substrates by microbial cells, that is, the catalytic reaction of specific groups of substrates by one or a series of enzymes produced in the process of microbial metabolism. It has become an effective tool widely used, especially for structurally complex natural products (Abourashed et al. 1999; de Carvalho and da Fonseca 2006). Microbial transformations can be considered as ‘‘green chemistry’’ for their ecological technology using mild conditions with less pollution. Additional benefits of biotransformation include its low cost, efficiency, strong chiral and stereoselectivity (de Carvalho and da Fonseca 2006), which are superior to conventional chemical synthesis methods. Microbial transformation is also an efficient way to mimic the mammalian metabolism (Abourashed et al. 1999). Previously, artemisinin (**1**) was transformed to various metabolites via deoxygenation (reduction) (Lee et al. 1989; Yu et al. 2017; Zhan et al. 2002b), hydroxylation (Gaur et al. 2014; Parshikov et al. 2006, 2004; Zhan et al. 2002a), oxidation (Liu et al. 2006), C-acetoxylation (Goswami et al. 2010), deoxygenation and hydroxylation (Lee et al. 1989; Ponnapalli et al. 2018), dihydroxylation (Zhan et al. 2017) and epoxylation (Zhan et al. 2015).

In this study, we report the biotransformation of artemisinin using a panel of microorganisms, further extraction, isolation, structural elucidation, kinetic monitoring and evaluation of in vitro anti-plasmodial activity of the transformation products.

## Materials and methods

### Chemicals

Artemisinin (98%) was purchased from Macklin Co., Ltd (China) which was used for biotransformation and bioactivity studies. HPLC grade formic acid, acetonitrile, hexane and ethyl acetate were purchased from Merck (Darmstadt, Germany). The other chemicals were purchased from Sinopharm Chemical Reagent Co., Ltd. The thin layer chromatography (TLC) plate was obtained from Qingdao Haiyang Chemical Group Co., People’s Republic of China.

### Microbial strains

*Aspergillus niger CICC 2487*, *Bacillus subtilis CICC 10157*, *Streptococcus thermophiles CICC 6038* and *Phanerochaete chrysosporium* *CICC 40719* were purchased from the China General Microbiological Culture Collection Center. *Trichoderma reesei ATCC 56765* and *Lactococcus lactis ATCC 11454* were purchased from the *American Type Culture Collection. Saccharomyces cerevisiae* is from Mauripan Co.

### Cultivation of microbial strains

The fungal strain *A. niger* was maintained on potato-dextrose agar plates at 4 ℃ and freshly sub-cultured before the transformation experiment. Fungal mycelia from agar slope cultures were transferred into 2-L Erlenmeyer flasks containing 1 L of potato dextrose liquid (PDL) medium, and incubated for 3 days at 180 rpm and 28 ℃ in a rotary shaker.

### The screening procedure for small scale of biotransformation of artemisinin with microbial strains

Fungal mycelia were moved into 100-mL shaker flasks containing 40 mL of different types of culture media depending on the microbial strains (Supplementary Materials). After 2 days of incubation at 28 ℃ and 180 rpm on a rotary shaker, 10 mL of cultures containing mycelia were transferred to inoculate 100 mL of medium in 250-mL Erlenmeyer flasks. The inoculated flasks were incubated for 2 days on rotary shakers at the same speed and temperature as before. One flask was kept as control without the addition of artemisinin. 2 mL of artemisinin in acetone at a concentration of 100 mg/mL was added into each flask. A total of 200 mg of substrate was used in the biotransformation, and the final concentration of artemisinin in the flask was 2 mg/mL. The cultures were incubated under the same conditions for an additional 14 days. The mycelia were separated by filtration or centrifugation and discarded. The filtrate or supernatant was extracted three times with ethyl acetate. The combined extract was evaporated under vacuum and under 40 °C using a rotary evaporator.

### Large scale of biotransformation of artemisinin with *A.niger*

*A. niger* mycelia were transferred into 100-mL Erlenmeyer flasks containing 40 mL of PDL medium for 3 days of incubation. The fermentation broth from the above Erlenmeyer flask was transferred into 2-L Erlenmeyer flasks containing 1 L of PDL medium with the same culture conditions. 20 mL of artemisinin (100 mg/mL in acetone) was added into each of the above flask with 1L-fermentation broth to achieve a final concentration of 2 mg/mL. The control was kept without addition of artemisinin. Substrate controls consisted of sterile medium containing the same amount of artemisinin and incubated under the same conditions. A total of 4 g of artemisinin was transformed with 2 L of fermentation broth. The cultures were maintained under the same conditions for 14 days. Later on, all fermentation broth was combined and the sediment was separated by centrifugation at 8500 *g* for 20 min. The supernatant (about 2 L) was extracted three times with ethyl acetate. The extract was evaporated under reduced pressure to afford a residue for further analysis and purification.

### LC-HRMS analysis of biotransformed products of artemisinin by *A.niger*

Liquid chromatography and high-resolution mass spectrometry (LC-HRMS) were applied for detecting the biotransformation products of artemisinin by the culture supernatant of *A. niger*. The re-dissolved transformation product was analyzed using LC-HRMS (Agilent 1290—Bruker micrOTOF QII, Bruker). The resolving power was 17,500 for full-scan and mass accuracy for 5 ppm. Chromatographic separation was achieved on an Agilent zorbax SB18 column (50 mm × 2.1 mm i.d., 1.8 µm). The LC–MS experiments were performed using a mobile phase A of 0.1% formic acid and a mobile phase B of acetonitrile containing 0.1% formic acid at a flow rate of 0.7 ml/min. The high-performance liquid chromatography (HPLC) gradient system for metabolites identification started with 30% A and linearly increased to 80% A in 5 min.

### Purification and isolation of biotransformation products by *A.niger*

The ethyl acetate extract (2.0 g) consisting of artemisinin transformation products was added to 4 mL of methanol, the precipitated solid was removed and identified as the untransformed artemisinin. The solution phase was evaporated and submitted for silica gel chromatography (50 g silica gel, 70 A size) eluting with hexane with increasing ethyl acetate (from 5–100%). 15 fractions were collected and the solvents were evaporated. The sub-fractions were further purified by semi-preparative high-performance liquid chromatography (HPLC) using Agilent 1220 LC (USA) system. The mobile phase A consisted of water and solvent B was methanol. The mobile phase calibration rose from 20% by 80% (A + B) over a period of 25 min to 100% B and kept at 100% for 10 min at a flow rate of 4 mL/min at 215 nm on semi-preparative HPLC column (Phenomenex, UK; 5 µm particle size: 9.4 × 250 mm). Four compounds: compound **2** (3 mg, 98% purity), compound **3** (25 mg, 95% purity), a mixture of compound **1** and **4** (1:1, 15 mg), and compound **5** (55 mg, 98% purity) were isolated from the extract of *A. niger*.

### Time-course biotransformation of artemisinin (1) monitored by ^1^H NMR spectroscopy

Each 100 mg of artemisinin dissolved in 2 mL acetone was transferred into ten Erlenmeyer flask containing 200 mL of medium with inoculation of *A.niger*. 100 mg of artemisinin in 2 mL acetone was also transferred to a flask containing only medium and incubated for 336 h. Cell culture of *A. niger* without addition of **1** was used as control. The transformation flasks were incubated and the extraction of transformation products at 48, 120, 192, 264 and 336 h in two replicates was performed as described above. The transformation products were identified by ^1^H NMR spectroscopy (400 MHz Bruker AVANCE NEO instrument) in CDCl_3_ in comparison with the ^1^H NMR data of **1–5** obtained before and that of **7** as reported (Zhan et al. 2015). The change of the percentages of the substrate **1** and the biotransformation products were plotted according to the integration values of the distinct peaks for each compound.

### NMR spectroscopy

1D and 2D NMR spectra of compounds **2**, **3**, **4** and **5** were obtained with a Bruker Ascend 400 NMR spectrometer (MA, USA). Bruker TopSpin 4.1.3 software or ACD/Labs 10 Freeware (Advanced Chemistry Development Inc., Ontario, Canada) was used to analyze the NMR spectra.

### Gas chromatography mass spectrometry

1–2 μL of the isolated compounds dissolved in ethyl acetate was injected into gas chromatography mass spectrometry (GC–MS) system consisting of an Agilent 7890 coupled to Agilent MS model 5975C MSD (Agilent Technologies, US). The GC started from150 °C increasing to 300 °C at the rate of 10 °C/min, which was then kept at 300 °C for 4 min with a constant helium pressure (10 psi). The mass spectra data were acquired in the scan mode in the m/z range 40–1000.

### High resolution electrospray mass spectrometry

The purified compounds **2**, **3**, **5**, and a mixture of **1** and** 4** were dissolved in methanol and injected to a LTQ Orbitrap mass spectrometer system (Thermo Scientific, MA, USA) to determine their high-resolution molecular masses and their fragment ions (MS/MS) using  a positive or negative electrospray ion source.

### Fourier transform infrared spectroscopy (FT-IR)

FT-IR instrument (Nicolet AVATAR360, Thermal Fisher, USA) was used to determine the functional groups of isolated compounds. FT-IR spectra were recorded from 4000 to 800 cm^−1^ using an attenuated total reflection (ATR) mode. The powder of isolated artemisinin derivatives were pressed against the ATR wafer.

Compound **2**: white powder. GC-EI-MS, *m/z* (%): 223.1 (1), 195.0 (1), 166.1 (80), 151.1 (92), 137.0 (100), 98.0 (40), 69 (35), 41.1 (50) (Figure S1). HRMS (positive ESI) *m/z* calcd for **2** ( [M + Na]^+^ 277.1416, found 277.1416 (Figure S2). HR MS/MS, 245.1152 [M -CH_3_OH]^+^. ART-FT-IR (ν_max_, cm^−1^): 3081, 2940, 2860, 1786, 1732, 1458, 1382, 1129, 992, 987. ^1^H, ^13^C NMR, and 2D NMR data (see Table 1 and Figure S4-9).

**Table 1:** ^1^H (400 MHz) and ^13^C NMR (100 MHz) data of compound **2** and **3** (in CDCl_3_)

H or C number	Compound 2					Compound **3**	
	^1^H NMR	^13^C NMR	H–H COSY	HMBC	NOESY	^1^H NMR	^13^C NMR
1	1.56 (1H, m)	57.4	H-2, H-9	H-13	H-2, H-9	1.47 (1H, m)	56.5
2	1.88 (2H, m)	27.6	H-1, H-3			1.50, 2.02 (each 1H, m)	26.9
3	3.81 (1H, dd); 4.11 (1H, ddd, 9.2, 7.7, 1.7 Hz)	69.4	H-2			4.15, 3.98 (2H, m)	67.2
4	5.17 (1H, s)	105.1		OCH_3_	OCH_3_	9.95 (1H, s)	205.3
5	1.56 (1H, m)	79.6		H-4, H-7		-	88.8
6	1.88 (1H, m)	46.8	H-7, H-10	H-12		1.95 (1H, m)	48.4
7	1.09, 1.90 (each 1H, m)	24.3	H6, H-8	H-6	H-6, H-8	1.87	27.4
8	1.07, 1.96 (each 1H, m)	35.2	H-7, H-9			1.10, 1.95 (each 1H, m)	35.1
9	1.67 (1H, m)	31.3	H-1, H-8, H-13	H-13		1.94 (1H, m)	30.6
10	3.06 (1H, qd, 9.2, 7.7 Hz)	34.7	H-6, H-12	H-12		2.51 (1H, m)	42.6
11	–	172.0	H-4, H10	H-4, H11, H-12		-	181.4
12	1.13 (3H, d, 8.0 Hz)	12.5	H-10			1.31 (3H, d)	17.2
13	0.94 (d, 3H, 8.0 Hz)	20.5	H-9			0.99 (3H, d)	20.4
OCH_3_	3.52 (3H, s)	55.1		H-4	H-4		

Compound **3**: white powder. GC-EI-MS, *m/z* (%): 211.1 (50), 165.1 (100), 137.1 (55), 109.1 (10), 89.0 (20), 41.1 (30) (Figure S10). HRMS (negative ESI) *m/z* calcd for [M-H]^+^ 239.1283, found 239.1182. ^1^H and ^13^C NMR data (see Table 1).

3-Hydroxy-deoxyartemisinin (**5**): white powder. HRMS (positive ESI) *m/z* calcd for [M + Na]^+^, 305.1365, found 305.1366. GC-EI-MS, *m/z* (%): 222.1 (70), 179.1 (30), 150.1 (55), 93.1 (40), 43.1 (100) (Figure S11). ART-FT-IR (ν_max_, cm^−1^): 3243, 2988, 2860, 1734, 1459, 1391, 1198, 1137, 985. ^1^H and ^13^C NMR data are consisted with those reported (Lee et al. 1989).

### Determination of antiplasmodial activity

Parasite strain Dd2^luc^ was maintained at 2% haematocrit, 0.5–5% parasitaemia and incubated at 37 °C in a gas chamber with 1% oxygen, 3% carbon dioxide and 96% nitrogen as described (Wong et al. 2011). Growth inhibition assays were carried out using a two-fold dilution of test compounds on 96-well plates, starting from 4 µM. A supralethal concentration (10 µM) of chloroquine was added to the negative (0% growth) control wells. The positive control (100% growth) wells were left untreated. *P. falciparum* culture (1–2% parasitaemia, 2% hematocrit) was added to all wells then incubated for 48 h at 37 °C in a gas chamber with 1% oxygen, 3% carbon dioxide and 96% nitrogen. A single-step lysis and bioluminescence assay to determine growth relative to control was carried out as previously described (Hasenkamp et al. 2012). IC_50_ values were estimated from nonlinear regression (sigmoidal concentration–response/variable slope equation) of log_10_-transformed concentration versus normalized growth (%) using GraphPad Prism v5.0 (GraphPad Software, Inc., San Diego, CA, USA).

## Results

### Structural elucidation of biotransformation products of artemisinin (**1**)

**Fig. 1:**
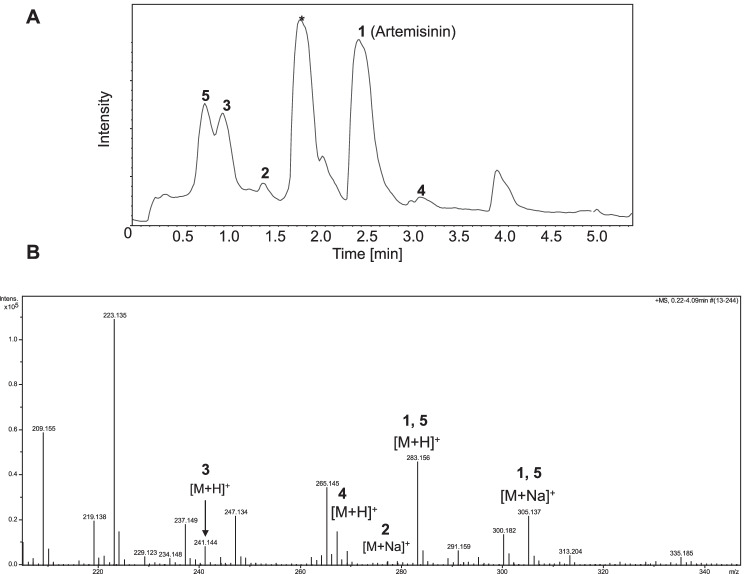
LC-HR-MS analysis of biotransformation products of artemisinin (**1**) by *A. niger*. **A**) Total ion chromatogram of high-resolution positive electrospray ionization mass spectrometry of the biotransformation products. **B**) Full scan mass spectrum of peaks from 0.5 to 4 min. HR-( +) ESI MS of peaks of **2**, **3**, **4** and 1/**5/7** showed the masses of 241.144 [M + H]^+^, 277.215 [M + Na]^+^, 267.172 [M + H]^+^ (289.154 [M + Na]^+^) and 283.155 [M + H]^+^ (305.138 [M + Na]^+^), respectively. A peak labelled by * showing a major mass of 223.136 is an unknown compound

A panel of seven microorganisms was screened for their capability to biotransform artemisinin by thin layer chromatography (TLC) and/or analytical high-performance liquid chromatography (HPLC) of the ethyl acetate extract of microbial cells after incubation with artemisinin (**1**). *Aspergillus niger* was found to be most efficient to produce different products and selected for preparative scale biotransformation with artemisinin, while others did not yield detectable products. TLC analysis (Figure S1) and HPLC demonstrated the formation of new products compared to the artemisinin and the control supernatant of *A. niger*. Further liquid chromatography (LC) high-resolution (HR) mass spectrometry (MS) analysis of the extract of *A. niger* after incubation with artemisinin (**1**) confirmed the formation of a number of transformation products (**2**–**5**) (Fig. 1).

**Fig. 2:**
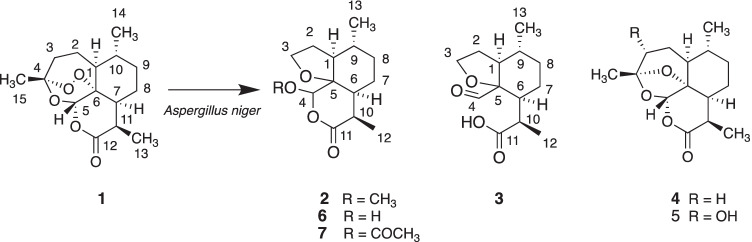
Chemical structures of biotransformation products (**2**–**5**) of artemisinin (**1**) by *A. niger*. Compounds **6** (Pandey et al. 2015) and artemisinin G (**7)** (Zhan et al. 2015) are analogues of compound **2**, previously identified as biotransformation products by plant-cultured cells and other microorganisms

A larger scale of transformation of artemisinin at gram scale with *A. niger* was performed to obtain more products. Using a combination of silica gel chromatography and semi-preparative HPLC, compounds **2**, **3** and **5** were isolated in high purity, while compound **4** was isolated as a mixture with recovered artemisinin (1:1). These compounds were identified using HR-ESI–MS, GC–MS, FT-IR, 1D and 2D NMR. Among them, compound **2** is a novel metabolite of artemisinin (Fig. 2).

Compound **2** was isolated as white powder with high purity (98%) as evidenced by GC–MS (Figure S2). High-resolution ESI–MS (Figure S3) determines the formula of **2** as C_14_H_22_O_4_ (found 277.1416, calculated for C_14_H_22_O_4_Na [M + Na]^+^, 277.1416 as sodium adduct) indicating the presence of four unsaturation units. This is consistent with the mass for compound **2** found in LC-HRMS (Fig. 1). FT-IR spectrum of **2** indicates the presence of an ester bond (1732 cm^−1^). ^1^H NMR (Figure S4), ^13^C NMR, DEPT (Figure S5) and HSQC (Figure S6) analysis indicates the presence of eight of a sum of CH and CH_3_, four methylene (CH_2_) and two quaternary carbons. There is an ester carbon (δ_c_, 172.0 ppm), three oxygenated carbons (δ_c_, 69.4, 79.6, and 105.1 ppm) and a methoxy group (δ_c_, 55.1 ppm). ^1^H NMR, HSQC and ^1^H-^1^H COSY (Figure S7) support the presence of the two methyl groups (δ_H_, 1.13 (3H, d, 8.0 Hz); 0.94 (3H, d, 8.0 Hz)) and a OCH_3_ group (δ_H_, 3.52 (3H, s)). HR ESI MS/MS showed a strong fragmentation ion at *m/z* 245.1152 [MCH_3_OH]^+^ after collision of ion at *m/z* 277.1512 [M + Na]^+^, which indicates a loss of CH_3_OH group (a mass difference of 32.0360).

Comparing the ^1^H and ^13^C NMR of **2** with those of compound **6**, 4-hydroxy-9,10-dimethyloctahydrofuro-(3,2-i)-isochromen-11(4H)-one (Table S1), which was reported as a biotransformation product of artemisinin in hairy root cell culture (Pandey et al. 2015), compound **2** has an extra OCH_3_ group, which suggests that **2** is a methyl ether of compound **6** at C4-OH position. The change of chemical shifts of neighboring carbons such as C-4, 5, 6, 10 and 11 also supports such structure. HSQC and ^1^H-^1^H COSY allow to complete the assignment of ^1^H and ^13^C NMR peaks (Table 1). Furthermore, the observation of the crossing spots between the peaks at 5.17 (H-4, s) and 3.52 (OCH_3_, s) on the NOESY spectrum (Figure S8) and the spot between the peak at *δ*_H_ 5.17 (H-4, s) and at 105.1 (C-4) on the HMBC spectrum (Figure S9) both support that the OCH_3_ group is attached to C-4 position. The ^1^H and ^13^C NMR data of compound **2** are also similar to those of artemisinin G (7) (Zhan et al. 2015), an acetylated compound **6** at position C-4 (Table S1). Therefore, compound **2** is identified as 4-methoxy-9,10-dimethyloctahydrofuro-(3,2-i)-isochromen-11(4H)-one, a new compound (Fig. 2).

Compound **3** was isolated as white powder. Both positive and negative ESI-HRMS confirmed its molecular formula as C_13_H_20_O_4_, suggesting to be a degradation product of artemisinin by loss of two carbons and an oxygen atom. ^1^H and ^1^^3^C NMR analysis indicates the presence of a carboxylic acid group, an aldehyde group and a furan ring. GC-EI-MS analysis of **3** (Figure S10) indicates the presence of a strong ion *m/z* at 211.1 due to loss of a mass of 29 (CHO) from its molecular ion *m/z* at 240.1. After comparison of ^1^H and ^13^C NMR data of **3** with those of compound **2** (Table 1), most of peaks associated with 6-membered and 5-membered tetrahydrofuran rings are similar except those for the lactone moiety in **2**; therefore, compound **3** is suggested to be the hydrolyzed form of 4-hydroxy-9,10-dimethyloctahydrofuro-(3,2-i)-isochromen-11(4H)-one (compound **6)** (Fig. 2).

Compound **4** was not separated from compound **1** by HPLC, but is identified as deoxyartemisinin by GC–MS and NMR data. Compound **5** was identified as 3-hydroxy-deoxyartemisinin by GC–MS (Figure S11), HR-ESI–MS, 1D and 2D NMR and comparison with literature data (Lee et al. 1989).

### Time-course of the biotransformation of artemisinin (**1**)

**Fig. 3:**
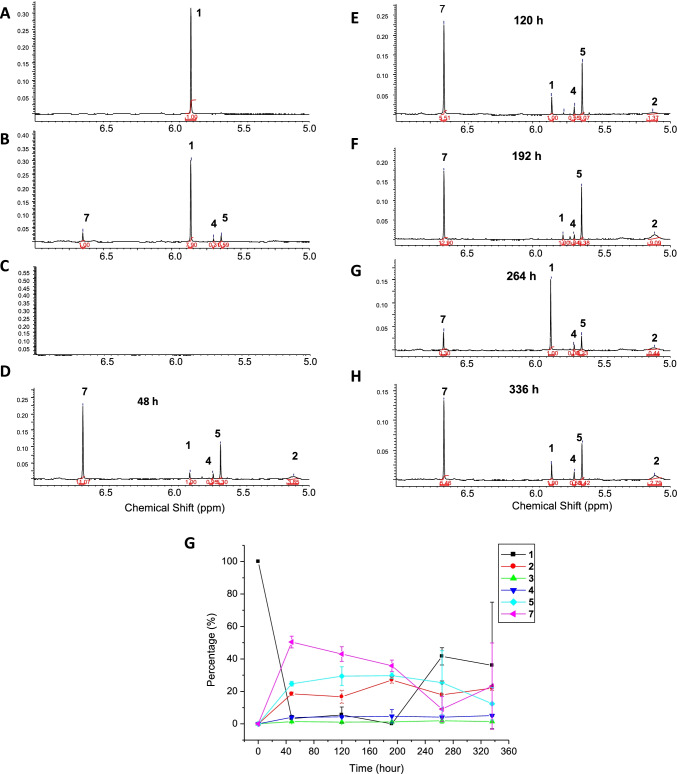
Kinetics of biotransformation of artemisinin (**1**) over 14 days (336 h) by ^1^H NMR spectroscopy. **A**) pure artemisinin. **B**) artemisinin **1** in the culture medium without *A. niger* over 336 h; most of the artemisinin remained except for the appearance of compounds **4** (3%), **5** (6%) and **7** (10%). **C**) metabolites of *A. niger* without the addition of **1**; no significant peaks between 5-7 ppm are present. **D**) incubation of **1** *with A. niger* for 48 h; **E**) incubation of **1** *with A. niger* for 120 h; **F**) incubation of **1** *with A. niger* for 192 h; **G**) incubation of **1** *with A. niger* for 264 h; H) incubation of **1** *with A. niger* for 336 h; I) The change of percentage of starting compound **1** and its biotransformation products (**2–5**, and **7**) over 336 h. The percentage of each compound at each time interval is calculated based on the integration value of the distinct singlet peaks at 5.88, 5.18, 9.95, 5.70, 5.64 and 6.66 ppm for the single proton in the respective compounds **1, 2, 3, 4, 5** and **7** (% of each compound represents as mean ± standard deviation (SD), n = 2)

To better understand the kinetics and pathways of the biotransformation of artemisinin (**1**) by *A niger*, the transformation products were monitored using ^1^H NMR spectroscopy (Fig. 3). In the control experiment, artemisinin could be slowly and chemically transformed to three compounds **4, 5** and **7** (yields less than 10%) in the medium at 30 ºC after 14 days without addition of *A. niger* (Fig. 3). When incubating **1** with *A niger* for 2 days, significant increase of **7**, **5** and **4** (in this order) were observed as well as the formation of compounds **2** and **3**. This indicated that certain enzymes from the fungi could catalyze these transformation processes. The levels of compounds **2**, **3**, **4** and 5 hardly changed from day 2 to day 14. Compound **7** decrease from day 2 to day 8, and artemisinin (**1**) was almost fully consumed on day 8. Interestingly and surprisingly, on day 11 and 14, artemisinin was found to be reformed and increased, while compound **7** was significantly decreased (Fig. 3B, Figure S12).

### Determining antiplasmodial activity of biotransformed artemisinin products

**Fig. 4:**
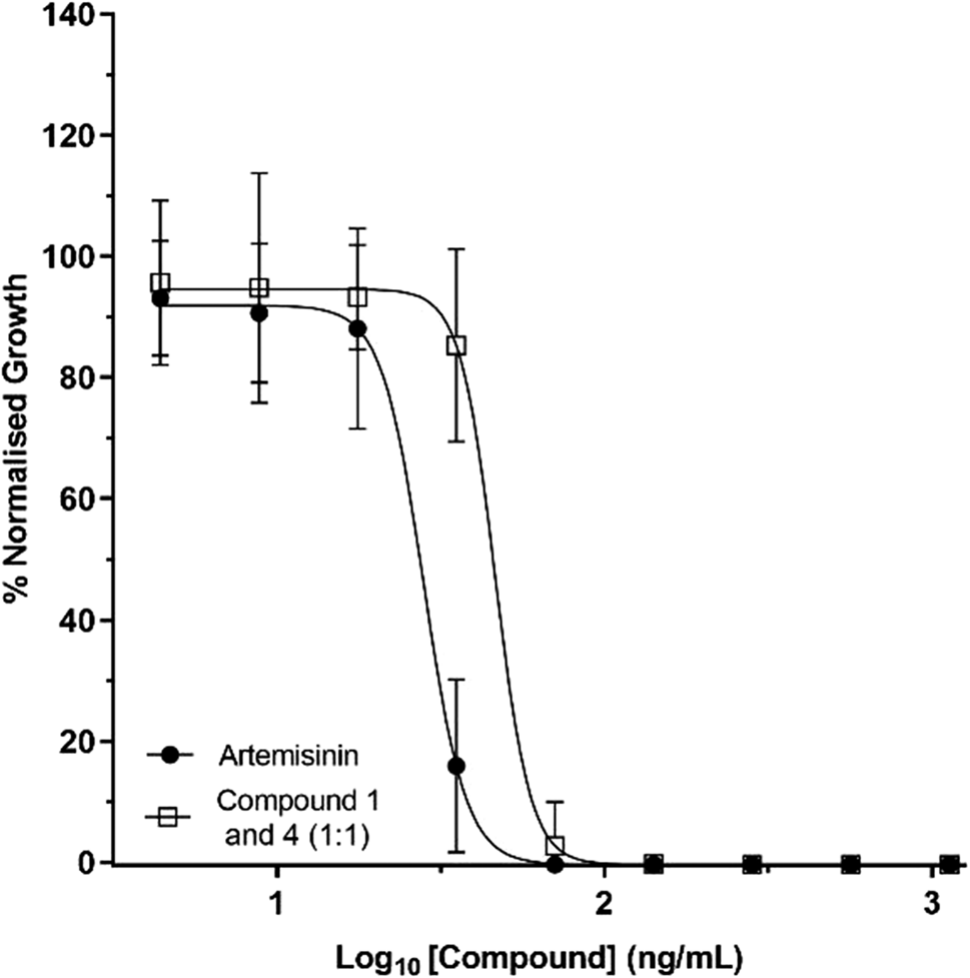
IC_50_ determination of artemisinin and transformed fractions. Log-transformed concentration versus a normalized % parasite growth (compared to untreated control) of *P. falciparum* Dd2^luc^ parasites after 48-h incubation with the indicated fraction. Data are presented as mean ± standard deviation with n =  ≤ 4 with a minimum of 2 biological repeats

The in vitro antiplasmodial activity of biotransformed artemisinin products was tested against intraerythrocytic stages of the *Plasmodium falciparum* chloroquine-resistant strain Dd2^luc^ (Ullah et al. 2020; Wong et al. 2011). Growth inhibition assays were carried out after 48-h incubation with each compound, with IC_50_ values estimated from data developed over three independent assays (Fig. 4). Compounds **2**, **3** and **7** were unfortunately not active (Table 2, Figure S13).

**Table 2:** In vitro antiplasmodial activity of artemisinin (**1**) and its biotransformed products

Compound	Antiplasmodial activity Yes/No	IC_50_ (ng/mL)
1	Yes	28.07 (95% CI 27.07–29.09)
1 and 4 (1:1)	Yes	46.36 (95% CI 44.52–48.27)
2	No	> 50,800
3	No	25,643 (95% CI 22,395 to 29,362)
5	No	> 53,200

## Discussion

Over the last three decades, extensive work has been carried out to make analogues of artemisinin using biotransformation with a range of microorganisms and plant cell cultures. The biotransformation of artemisinin (1) by *Nocardia corallina* (ATCC 19,070) and *Penicillium chrysogenum* (ATCC 9480) to deoxyartemisinin and 3- α-hydroxydeoxyartemisinin, respectively, was first reported in 1989 (Lee et al. 1989). Artemisinin biotransformation by *Cunninghamella echinulata* afforded 10-β-hydroxyartemisinin (Zhan et al. 2002a)*,* and by *C. elegans* yielded 7-β-hydroxyartemisinin as the major product along with other minor bioconversion products: 7-β-hydroxy-9-α-hydroxy-artemisinin, 4-α-hydroxy-1-deoxoartemisinin, 6-β-hydroxyartemisinin (Parshikov et al. 2004), 7-α-hydroxyartemisinin and 6-β-7-α-dihydroxyartemisinin (Zhan et al. 2017). Microbial metabolism of artemisinin by *Mucor polymorphosporus* resulted in the production of 9-β-hydroxyartemisinin, 3-β-hydroxyartemisinin and deoxyartemisinin (Zhan et al. 2002b). Artemisinin (1) was converted to 5-β-hydroxyartemisinin and 7-β-hydroxyartemisinin by *Eurotium amstelodami* (Parshikov et al. 2006). Biotransformation of artemisinin by fungi *Rhizopus stolonifer* afforded a new compound, 1-α-hydroxyartemisinin and 10-β-hydroxyartemisinin and deoxyartemisinin (Gaur et al. 2014). Artemisitone-9, a ketone derivative of artemisinin, was produced by cultured *Streptomyces griseus* ATCC 13,273 with artemisinin (Liu et al. 2006). Artemisinin was biotransformed to C-9 acetoxy artemisinin using *Penicillium simplissimum* along with C-9 hydroxy derivative (Goswami et al. 2010).

The fungi like *Aspergillus* showed to be an efficient machinery to metabolize artemisinin. Artemisinin was converted to deoxyartemisinin, 3-α-hydroxydeoxyartemisinin and 1-α-hydroxydeoxyartemisinin by *Aspergillus niger* AS 3.1858 (Zhan et al. 2002b). Further biotransformation of artemisinin (**1**) by *A. niger* resulted in 5-β-hydroxyartemisinin and 7-β-hydroxyartemisinin (Parshikov et al. 2006) similar as by *E. amstelodami*, and two novel compounds, 3-β-hydroxy-4,12-epoxy-1-deoxyartemisinin and 3,13-epoxyartemisinin together with known artemisinin G (**7**) and 3-α-hydroxydeoxyartemisinin by *A. niger* VKM F-1119 (Zhan et al. 2015). *A. terreus* transformed artemisinin to deoxyartemisinin and 4-α-hydroxy-1-deoxyartemisinin (= 3-α-hydroxydeoxyartemisinin) (Yu et al. 2017). The filamentous fungus *A. flavus* MTCC-9167 converted artemisinin to a new hydroxy derivative, 14-hydroxydeoxyartemisinin along with known deoxyartemisinin, artemisinin G (**7**) and 3-α-hydroxydeoxyartemisinin (Ponnapalli et al. 2018).

**Fig. 5:**
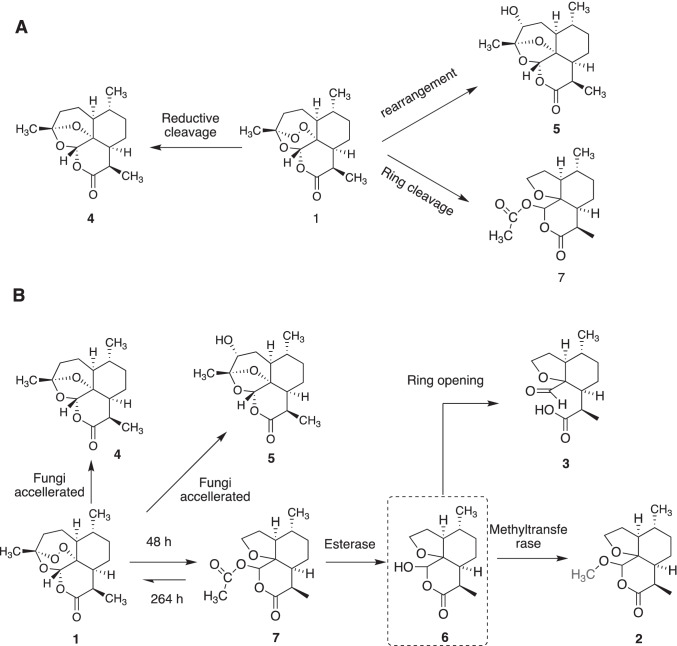
Proposed transformation pathways for the generation of compounds (**2**–**5**) from artemisinin (**1**) by the cell culture medium (A) and *A. niger* CICC 2487 (B), respectively. Compounds **4**, **5** and **7** were slowly formed due to inherent instability of artemisinin in the aqueous medium. The formation of these compounds were accelerated by *A. niger*, while also generating a new compound **2** via a possibly short-lived intermediate **6** from the initial product **7** together with the formation of compound **3**

In this study, a novel metabolite (**2**) of artemisinin was identified, which is structurally similar to artemisinin G (**7**), previously found from a biotransformation product of artemisinin by *A. niger* VKM F-1119 (Zhan et al. 2015). The strain of *A. niger* CICC 2487 used in our study may produce different enzymes compared to other *A.niger* strains, thus leading to the formation of the novel product **2**. The possible biotransformation pathway of artemisinin by *A. niger* CICC 2487 is proposed in Fig. 5. Artemisinin was found to be unstable in the culture PBL media and could be transformed to compound **7**, **5** and **4** (Fig. 5A). The change of artemisinin (**1**) to compound **4** in the culture medium was reported before (Zhan et al. 2002b). The thermal degradation of artemisinin to compounds **5** and **7** at a high temperature of 190 ºC for 10 min was also reported (Lin et al. 1985). The reducing agents such as Fe (II) could also lead to the formation of compounds **4**, **5** and **7** (Kapetanaki and Varotsis 2001; Wu et al. 1998). Our results further indicated the sensitivity and instability of artemisinin in agreement with these previous reports. However, this process was hugely accelerated by *A. niger* with further generation of compounds **2** and **3** (Fig. 5B). Artemisinin G (**7**) was initially formed via ring rearrangement of artemisinin (**1**) as also found previously by *A. niger* (Zhan et al. 2015), whose acetyl group could be subsequently hydrolyzed by an esterase to form compound **6** with a free hydroxy group, which was then methoxylated by a methyltransferase to form compound **2**. However, compound **6** was not detected by ^1^H NMR spectroscopy,  probably due to a very fast conversion step, which suggested the presence of an active esterase and methyltransferase allowing the full conversion of the intermediate **6** to the final and stable product **2**. Our kinetic study by ^1^H NMR spectroscopy (Fig. 3B) supported this proposed pathway. An interesting and surprising observation is that artemisinin was reformed after 11 days with a significant loss of artemisinin G (**7**), suggesting that on day 8 the fungus *A niger* might produce an unique enzyme allowing the convertion of the degradation product (**7**) back to the original artemisinin via unususal ring closure and rearrangement reactions. This is somehow consistent with our large scale of biotransformation of artemisinin, where a portion of artemisinin was recovered over the purification process while compound **7** was not isolated at all. The possible enzymes involved and associated mechanisms are under further investigation.

Compound **3** is also a degradation product of artemisinin with the loss of two carbons, which has not been found through biotransformation by either *A.niger* or other microbes but through thermal degradation at a high temperature of 180 °C (Xuan-De Luo et al. 1985). This is the first report of the production of **3** via mild biotransformation by a fungus. Compounds **4** and **5** were found in this study, in agreement with previous findings in the biotransformation of artemisinin by *A. niger* (Yu et al. 2017; Zhan et al. 2002b, 2015). Compounds **4** and 5 were suggested to be formed simultaneously and independently as happened at a high temperature (Lin et al. 1985) by reductive cleavage of the peroxide bridge of artemisinin (Fig. 3 and 5). This process of forming **4** and **5** seems to be different from the plausible biotransformation pathway of artemisinin by *Aspergillus flavus* (Ponnapalli et al. 2018) where compound **5** was suggested to be formed from compound **4** by hydroxylation reaction.

The IC_50_ of the artemisinin (**1**) was 28.1 ng/mL with the antiplasmodial activity almost completely ablated following biotransformation for compounds **2**, **3** and **5**. Whilst one fraction has antiplasmodial activity at a reduced potency (IC_50_ value of 46.2 ng/mL) compared to the artemsininin, this fraction contains both compound **1** (artemisinin) and compound **4** as a 1:1 mix. This suggests that the antiplasmodial activity of this fraction likely results from the artemsisinin content, and that compound **4** has no or almost none antiplasmodial activity. The antiplasmodial activity data is in agreement with repored data for compound **4** and **5** (Ponnapalli et al. 2018) and are not unexpected as the antiplasmodial activity of artemisinin and its active derivatives is attributable to the peroxide-containing moiety. In the presence of ferous ions (FeII), this moiety is cleaved to produce carbon and oxygen centred radicles (O'Neill et al. 2010). Following the biotransformation of artemisinin (**1**), this peroxide-containing moiety was removed and thus antiplasmodial activity lost. Nevertheless, the biotransformaton produced new molecular entities which are not easily accessible by chemical synthesis. Further derivation of these derivatives as precursors using chemical or biological means and additional biological activities including cytotoxicty against cancer are worth investigating. Our studies along with others indicated that *A. niger* could produce mainly the deoxyartemisinin derivatives, which can cause the significant loss of antiplasmodial activity. However, fortunately other microorganisms such as *Mucor polymorphosporus* (Zhan et al. 2002b), *Cunninghamella echinulata* (Zhan et al. 2002a)*, C. elegans* (Parshikov et al. 2004), *Eurotium amstelodami* (Parshikov et al. 2006) and *Rhizopus stolonifer* (Gaur et al. 2014) could yield (di)hydroxylation derivatives of artemisinin while retaining the crucial and sensitive peroxide bridge group for their antiplasmodial activity. In the future, those derivatives and novel hydroxy derivatives will be made using the same or related microorganism species by further targeted screening and tested for their likely improved activity. The hydroxylation artemisinin derivatives generated from microbial transformation may provide advantages over artemisinin itself by increasing water solubility and binding affinity to its targets. Conjugation of artemisinin with (iso)quinoline and other compounds into hybrid compounds has been demonstrated to be an effective way to tackle multidrug-resistant malaria (Capci et al. 2019; Peter et al. 2021). Therefore, making novel hybrid molecules based on the hydroxylation artemisinin derivative with a reactive hydroxy group and other antimalarial compounds such as (iso)quinonline (Capci et al. 2019), gallic acid (Aldulaimi et al. 2017), thymoquinone (Johnson-Ajinwo et al. 2018) and cycleanine derivatives (Uche et al. 2021, 2018) with different mechanisms of action could be a promising approach to attack artemisinin resistance.

In conclusion, a novel atemisinin derivative (**2**) along with three known compounds has been generated using mild microbial transformation of atemisinin with a strain of *A. niger* CICC 2487, whose structure was elucidated using extensive spectroscopic analysis. The antiplasmodial activity of all the transfomed products signficantly decreased due to the loss of the peroxide moiety in artemisinin. Hydroxylation artemisinin derivatives retaining a peroxide bridge through microbial transformation should be the future focus, which will allow facile chemical conjugation of artemisinin with other antimalarial compounds to tackle artemisinin-sensitive or artemisinin-resistant malaria through dual or multiple mechanisms.

## Supplementary Information

Below is the link to the electronic supplementary material.

Supplementary file1 TLC analysis, GC–MS, NMR data and IC_50_ curves against *P*. *falciparum* Dd2^luc^ parasites of artemisinin transformation products are available (PDF 522 KB)

## Data Availability

All data generated or analyzed during this study are included in this published article (and its supplementary information files).
