# Supplementary material for: Biotransformation of artemisinin to a novel derivative via ring rearrangement by Aspergillus niger
Source: Appl Microbiol Biotechnol. 2022 Mar 31;106(7):2433–44. doi: 10.1007/s00253-022-11888-0 (PMC8989930; doi:10.1007/s00253-022-11888-0)
Supplement: Supplementary file 1 — Supplementary file1 TLC analysis, GC–MS, NMR data and IC50 curves against P. falciparum Dd2luc parasites of artemisinin transformation products are available (PDF 522 KB) [file 253_2022_11888_MOESM1_ESM.pdf]

Applied Microbiology and Biotechnology

Supplementary Materials

Biotransformation of Artemisinin to a Novel Derivative via Ring Rearrangement by *Aspergillus niger*

Jiaer Luo,[1] Rebbeca Mobley[2], Sian Woodfine[3], Falko Drijfhout[3], Paul Horrocks[2], Xiaodong Ren[1,*], Wen-Wu Li[4, *]

[1] Department of Biopharmacy, School of Life Science, Jilin University, Changchun,130012, China

[2] School of Medicine, Keele University, Staffordshire, ST5 5BG, United Kingdom

[3] Chemical Sciences Research Centre, Keele University, Staffordshire, ST5 5BG, United Kingdom

[4] School of Pharmacy and Bioengineering, Keele University, Stoke-on-Trent, ST4 7QB, United Kingdom

Experimental

The bacteria *L.lactis* was maintained on MRS agar plates (peptone 10 g/L, meat extract 8 g/L, Yeast extract 4 g/L, glucose 20 g/L, sodium acetate·$3H_2O$ 5 g/L, Tween 80 1ml/L, Dipotassium hydrogen phosphate 2 g/L, triammonium citrate 2 g/L, magnesium sulfate·7 H_2O 0.2 g/L, manganese sulfate·4 H_2O 0.05 g/L) at 4℃ and freshly sub-cultured before using in the transformation experiment. Colony of bacteria from agar slope cultures were transferred into 250 mL Erlenmeyer flasks containing 100 mL of MRS medium for 48 h of incubation at 37 ℃ and 180 rpm in a rotary shaker.

The bacteria *B. subtilis* was maintained on BSM agar plates (peptone 5 g/L, beef extract 0.5 g/L, glucose 20 g/L, sodium chloride 5 g/L) at 4℃ and freshly sub-cultured before using in the transformation experiment. Colony of bacteria from agar slope cultures were transferred into 250 mL Erlenmeyer flasks containing 100 mL of BSM medium for 48 h of incubation at 37℃ and 180 rpm in a rotary shaker.

The bacteria *S.thermophiles* was maintained on M17 agar plates (phytopeptone 5 g/L, Yeast extract 5 g/L, beef extract 2.5 g/L, Polypeptone 5 g/L, β- Disodium glycerophosphate 19 g/L, MgSO4·7H2O 0.246 g/L) at 4℃ and freshly sub-cultured before using in the transformation experiment. Colony of bacteria from agar slope cultures were transferred into 250 mL Erlenmeyer flasks containing 100 mL of M17 medium for 48 h of incubation at 42℃ and 180 rpm in a rotary shaker.

The *S. cerevisiae* strains was maintained on YPD agar plates (peptone 20 g/L, Yeast extract 10 g/L, glucose 10 g/L) at 4℃ and freshly sub-cultured before using in the transformation experiment. The *S.cerevisiae* from agar slope cultures were transferred into 250 mL Erlenmeyer flasks containing 100 mL of YPD medium for 48 h of incubation at 30℃ and 180 rpm in a rotary shaker.

The fungal strains *T.reesei* or *P.chrysosporium* were maintained on potato-dextrose agar plates at 4℃ and freshly sub-cultured before the transformation experiment. Fungal mycelia

from agar slope cultures were transferred into 100 mL Erlenmeyer flasks containing 40mL of PDL medium for 72 h of incubation at 28℃ and 180 rpm in a rotary shaker. The fermentation broth from above Erlenmeyer flask was transferred into 2 L Erlenmeyer flasks containing 1 L of PDL medium for the same culture condition. The PDL medium was made by 200-300 g potatoes which were cut to pieces and added to deionized water. Then it was boiled and filtered. 20 g glucose was added into the supernatant, and then volume to 1000 mL with deionized water.

TLC analysis of biotransformation products of Artemisinin

Thin-layer chromatography (TLC) was applied for detecting the biotransformation products of artemisinin by the culture supernatant of *Aspergillus Niger*. The transformation products were re-dissolved in chloroform. TLC were carried out using a silica gel G plate and a developing solvent consisting petroleum ether, acetone and glacial acetic acid（8:2:0.1, v/v/v, where artemisinin and its biotransformation products on the plates were stained by spraying with 2% (v/v) vanillin and 20% (v/v) sulphuric acid in ethanol, and then heating at 85°C for 15 min until the spots are clear under visible light.

Analytical high performance liquid chromatography (HPLC)

10-20 µL of 1.0 mg/mL of biotransformed products dissolved in methanol was injected to analytical HPLC system. The mobile phase rose from 20% B (A + B) to 100% B over a period of 25 min and kept at 100% for 6 min at 215 nm on an analytical HPLC column (Phenomenex, UK; 5 µm particle size, 4.6 × 250 mm) at a flow rate of 1 mL/min. Solvent A consisted of 0.1 % TFA in water and solvent B was 80% acetonitrile with 0.1% TFA.

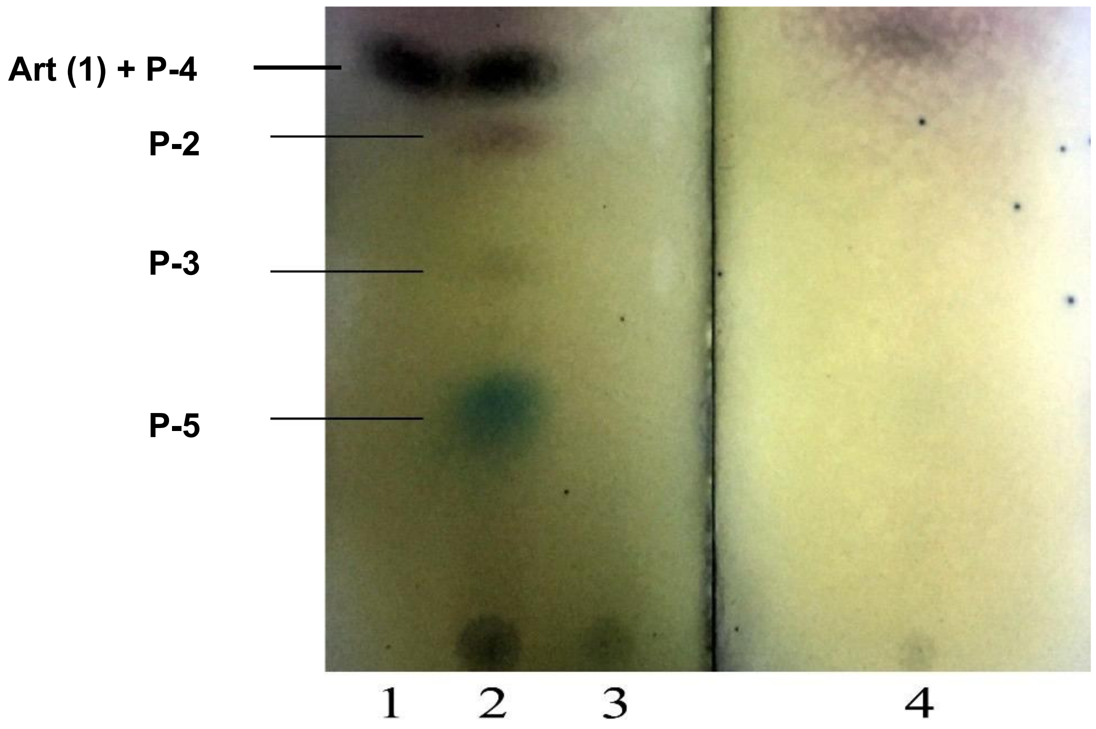

Figure S1. TLC analysis of artemisinin biotransformation products. **Lane 1**: artemisinin standard sample. **Lane 2**: the extract of *A.niger* culture supernatant added with the artemisinin standard sample, after 7 days of incubation, three major biotransformation products, p-**2**, p-**3** and p-5 were observed. **Lane 3**: the culture supernatant extract without artemisinin, after 7 days of incubation. **Lane 4**: sterile medium containing artemisinin, after 7 days of incubation.

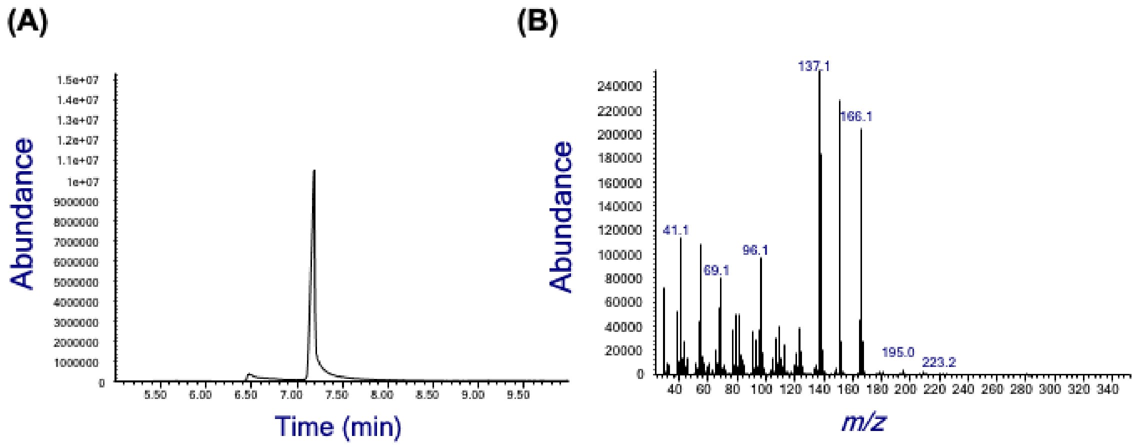

Figure S2. Gas chromatography (A) and electron ionization mass spectrum (B) of the isolated biotransformation product (**2**) of artemisinin.

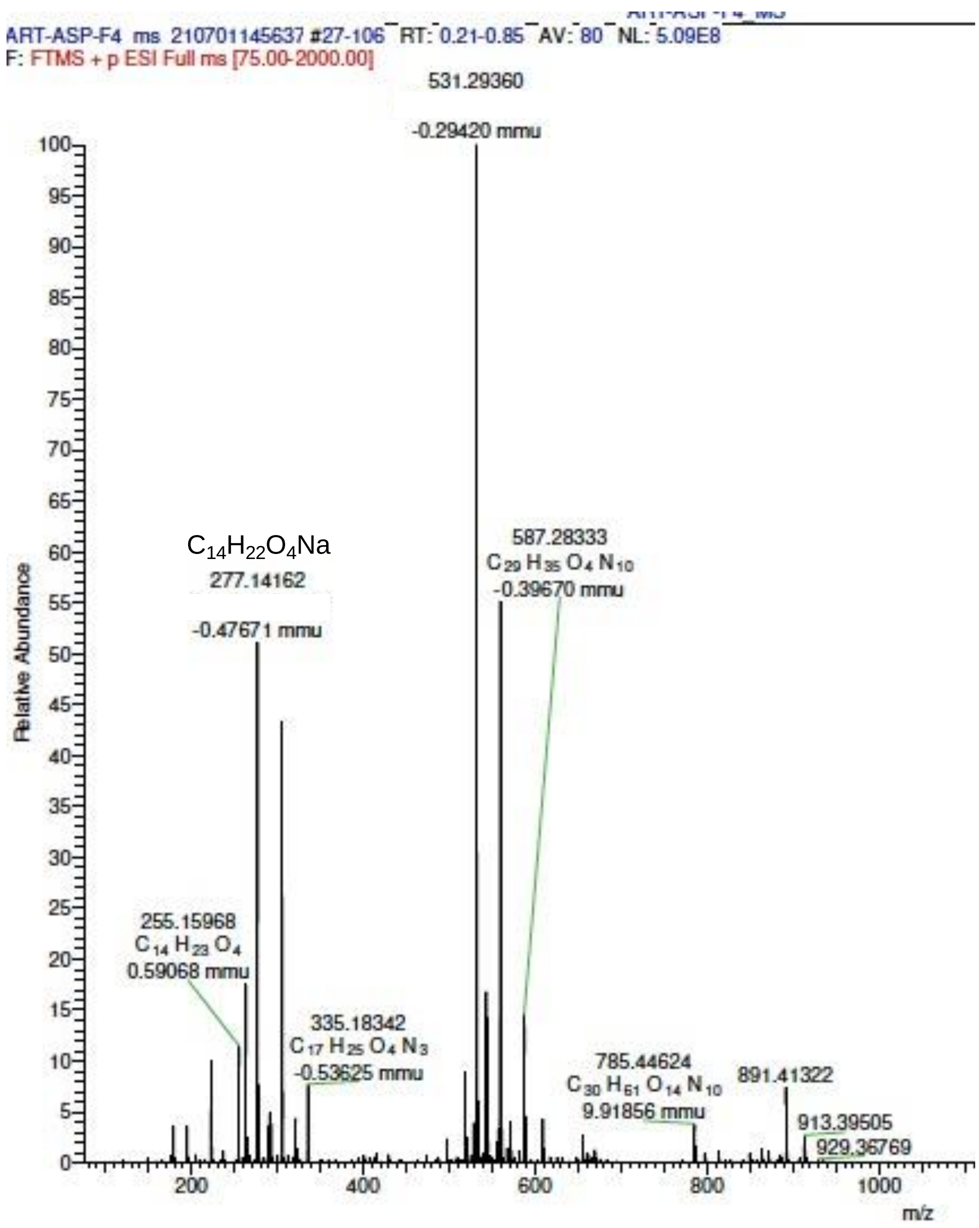

Figure S3. Positive mode HR ESI-MS spectrum of artemisinin derivative **2**.

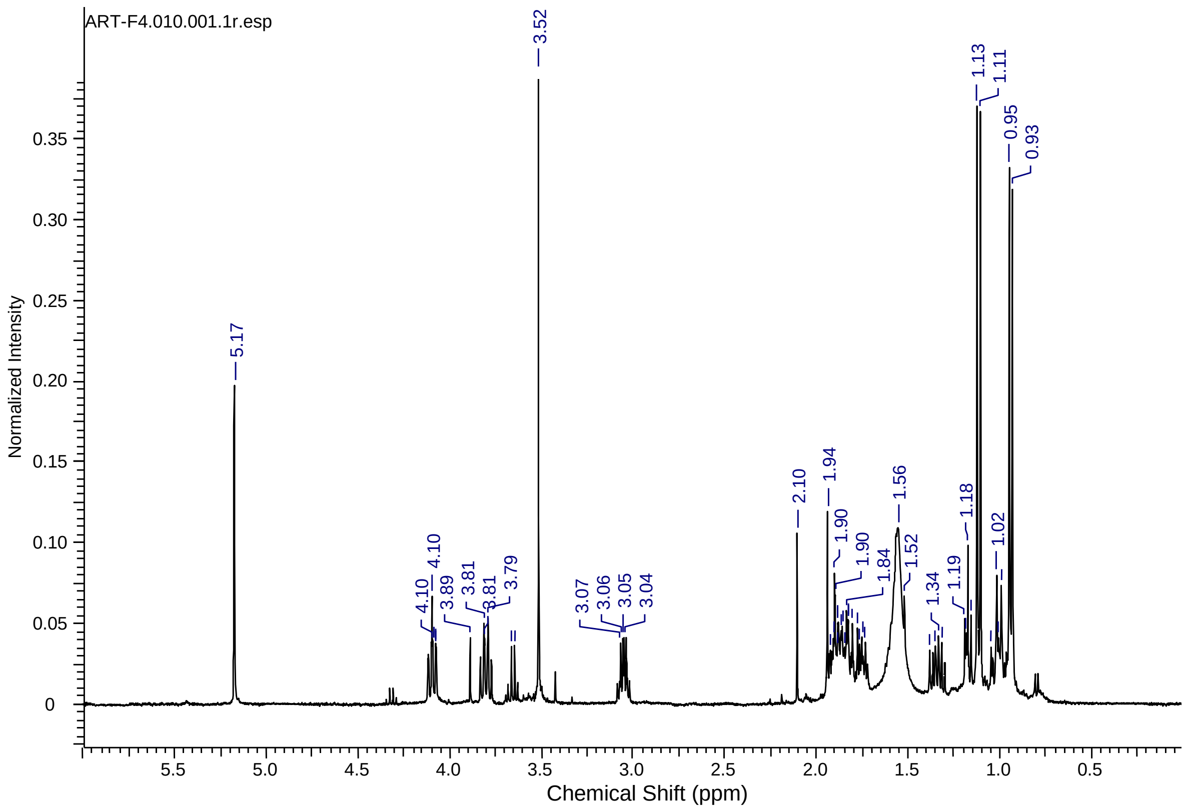

Figure S4. ^{1}H NMR spectrum (CDCL$_3$, 400 MHz) of compound **2**.

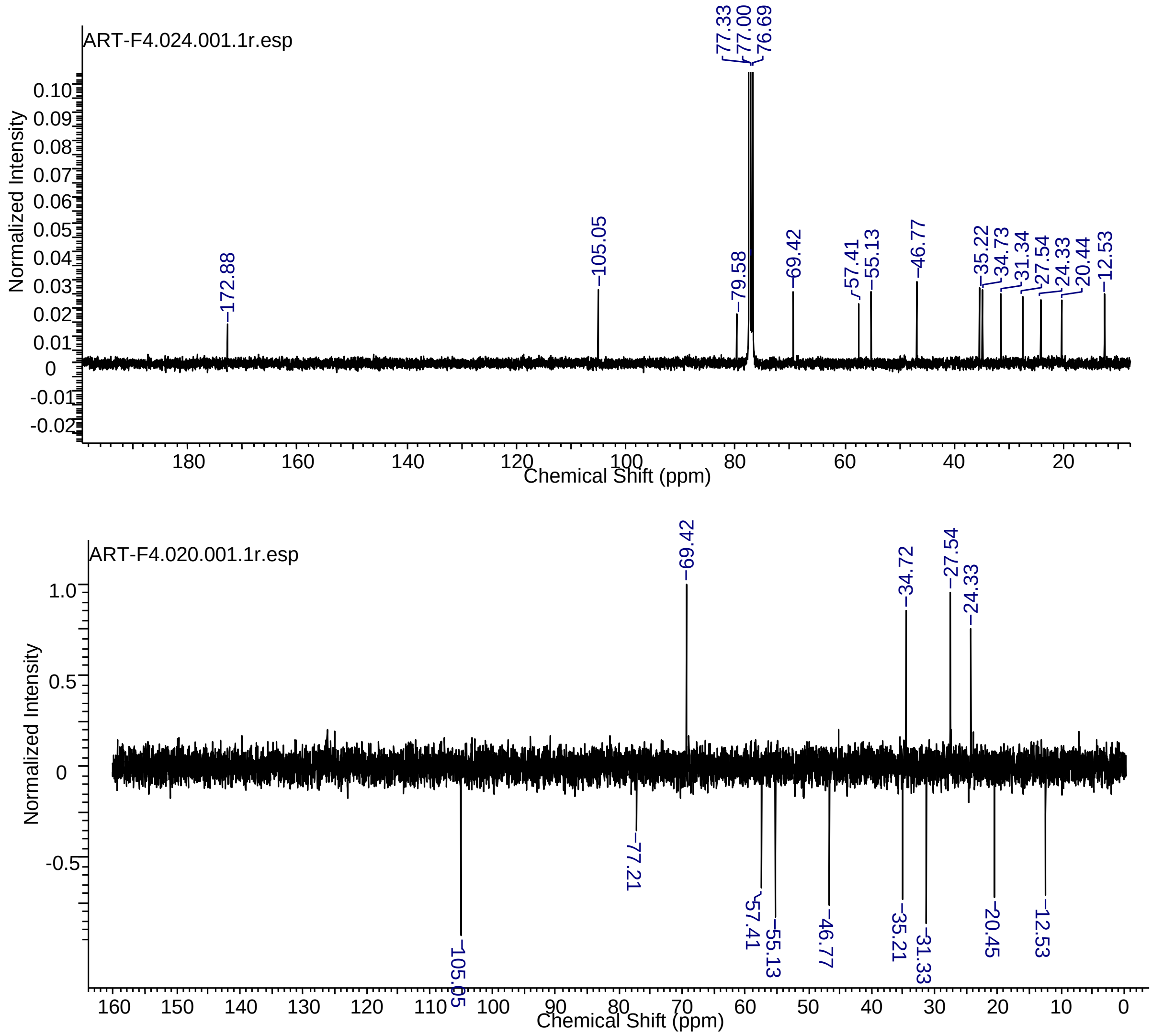

Figure S5. ^{13}C NMR and DEPT spectra ($CDCL_3$, 100 MHz) of compound **2**.

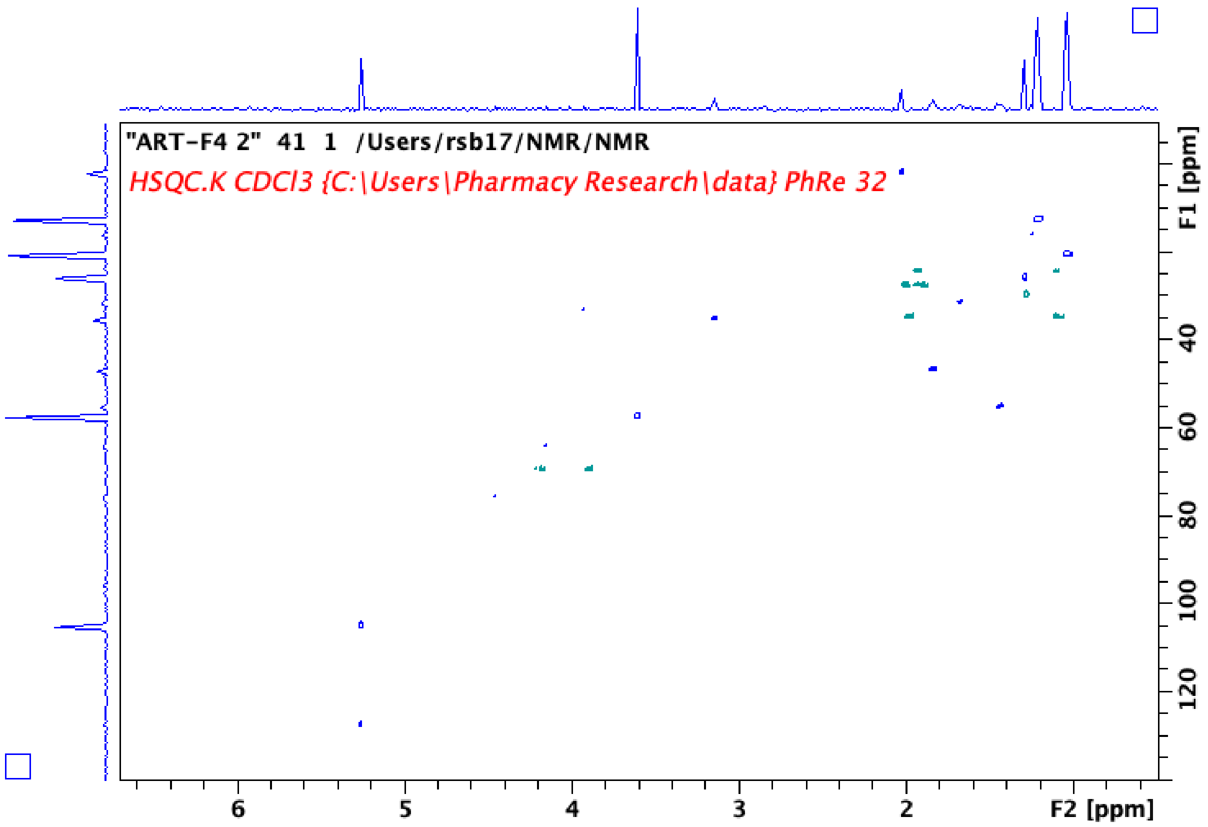

Figure S6. HSQC spectrum ($CDCl_3$, 400 MHz) of compound **2**.

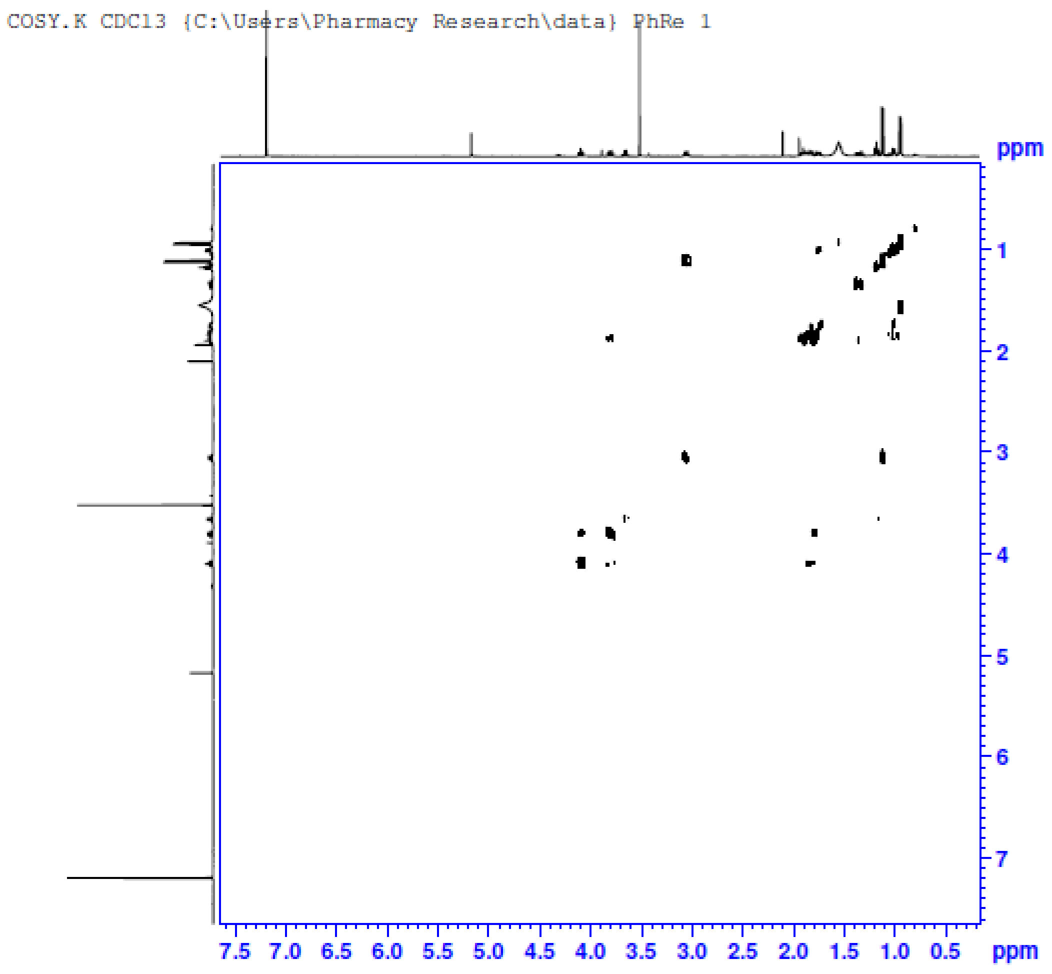

Figure S7. H-H COSY spectrum ($CDCl_3$, 400 MHz) of compound **2**.

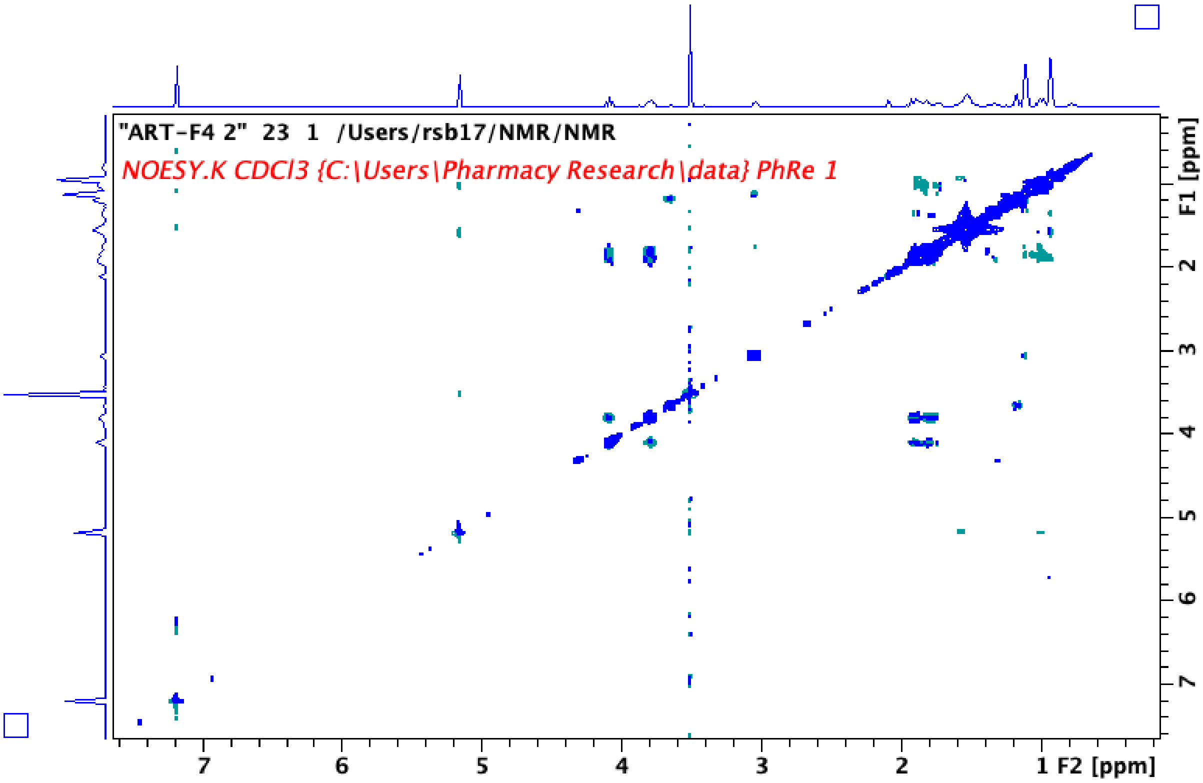

Figure S8. NOESY spectrum (CDCL$_3$, 400 MHz) of compound **2**.

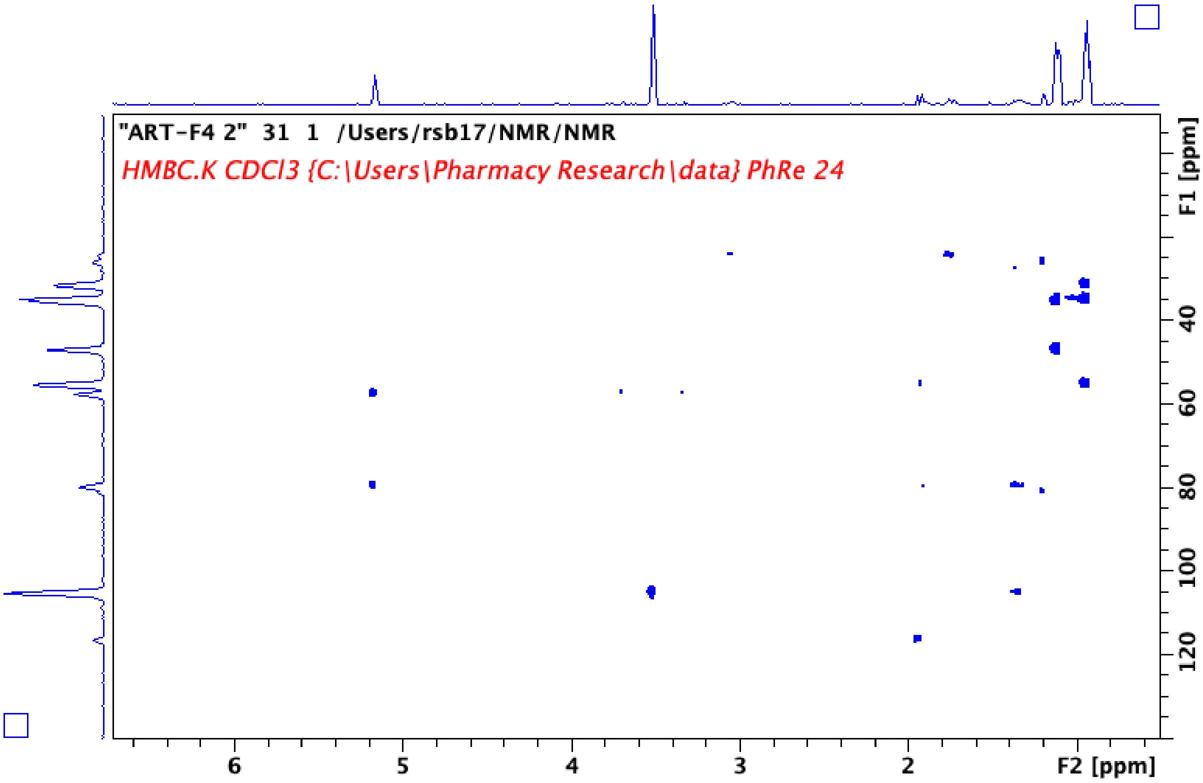

Figure S9. HMBC spectrum ($CDCl_3$, 400 MHz) of compound **2**.

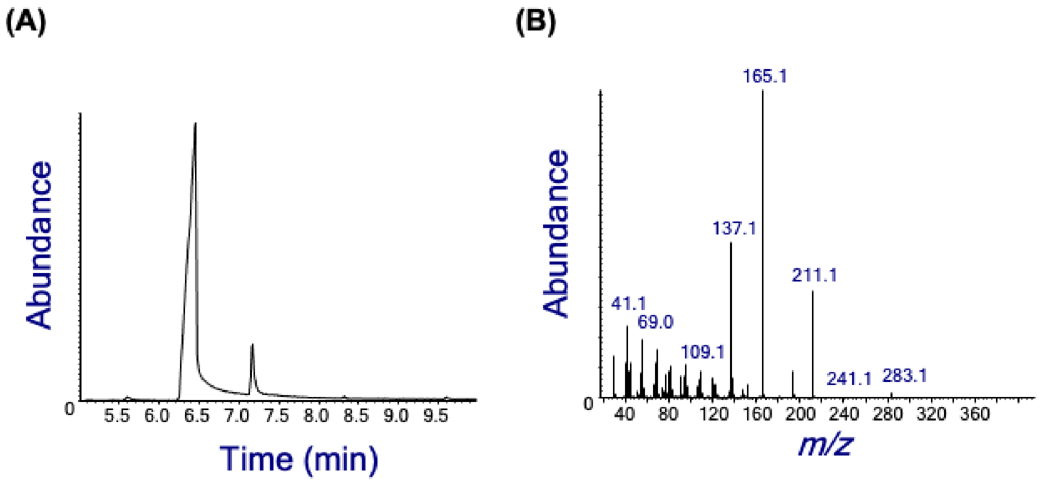

Figure S10. Gas chromatography (A) and MS spectrum (B) of biotransformed product (**3**) of artemisinin.

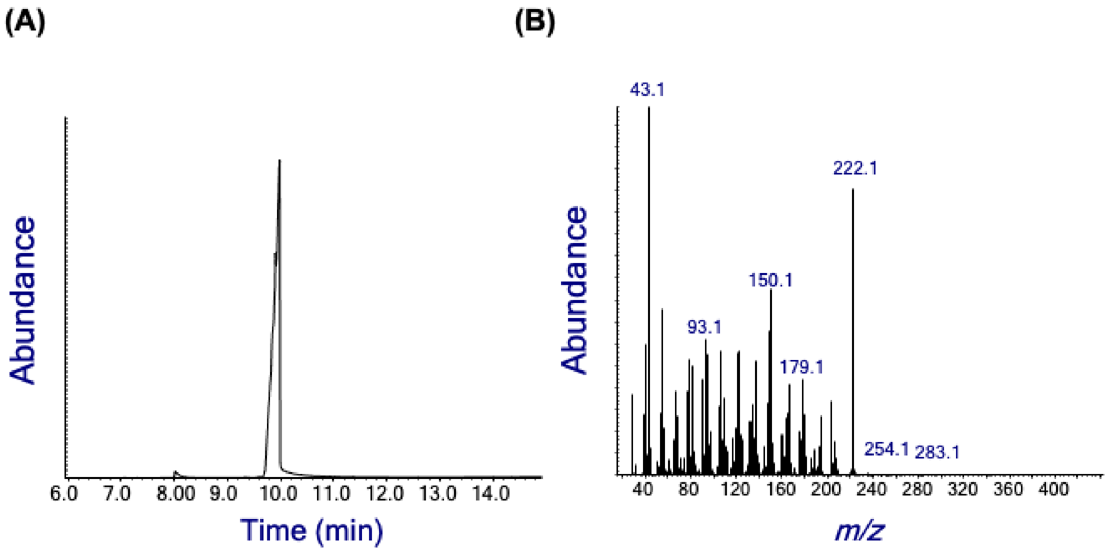

Figure S11. Gas chromatography (A) and EI mass spectrum (B) of biotransformation product (**5**) of artemisinin. The purity of compound **5** is 98%.

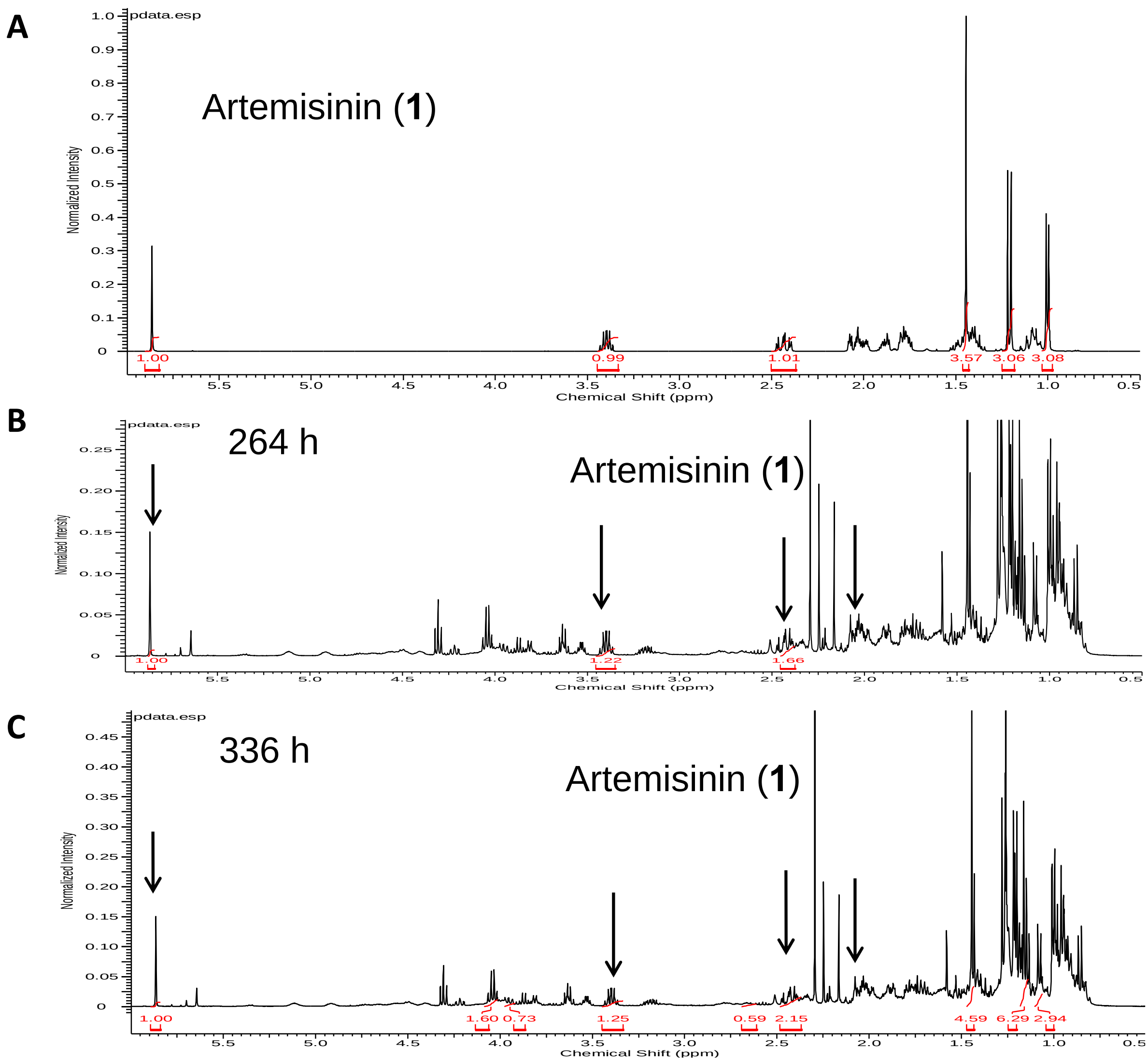

Figure S12. ^{1}H NMR (400 MHz, $CDCl_3$) spectra of artemisinin (A), its biotransformation products at 264 h (B) and at 336 h (C). B and C clearly indicated the reappearance and significant increase of artemisinin 1, which was almost undetectable at 192 h (Figure 3).

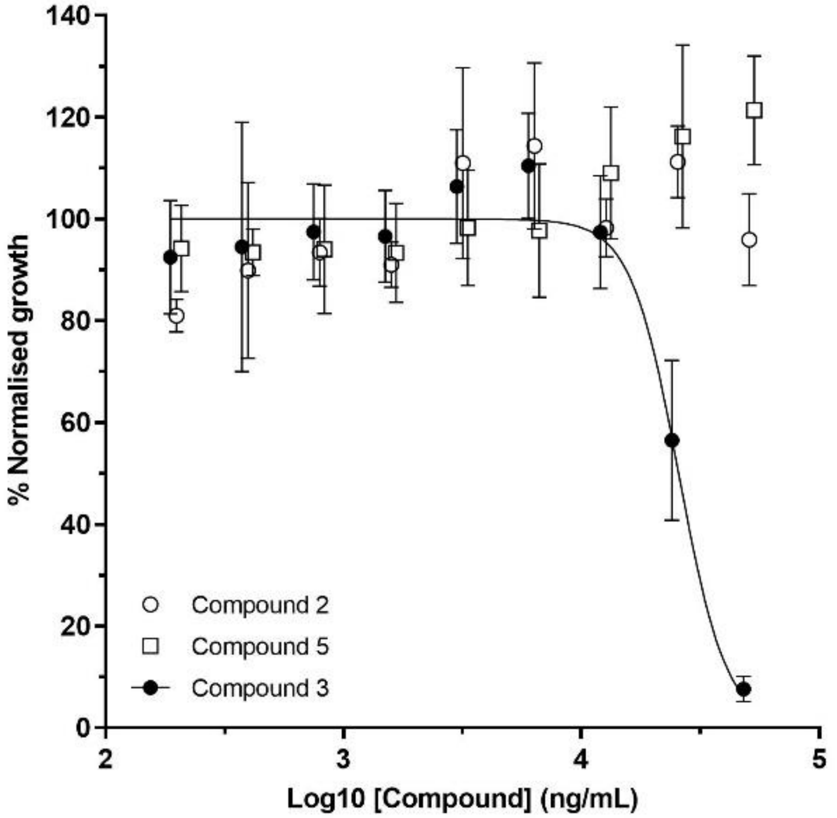

Figure S13. IC_{50} determination of biotransformation products **2, 3** and **5**. Log-transformed concentration versus a normalised % parasite growth (compared to untreated control) of *P. falciparum* $Dd2^{luc}$ parasites after 48-hour incubation with the indicated fraction. Data is presented as mean ± standard deviation with n= ≤ 4 with a minimum of 2 biological repeats.

Table S1. Comparison of ^{1}H (400 MHz) and [13] C NMR (100 MHz) data of compound **2** with reported data of compounds **6** and 7 (all in $CDCl_3$).

	**2**		**6** [1]		**7 (artemisinin G)** [2]	
H or C number	^{1}H NMR	^{13}C NMR	^{1}H NMR	^{13}C NMR	^{1}H NMR	^{13}C NMR
1	1.56 (1H, m)	57.4	1.54 (1H, m)	56.3	1.47 (1H, m)	54.6
2	1.34, 1.88 (each 1H, m)	27.6	1.43, 1.88 (each 1H, m)	27.4	1.57, 2.02 (each 1H, m)	27.5
3	4.11 (1H, dd, 9.2, 7.7 Hz); 3.81 (1H, dd);	69.4	4.15 (1H, dd); 3.96 (1H, dd);	68.3	4.20 (1H, t, 8.4 Hz);, 3.92 (1H,m)	69.1
4	5.17 (1H, s)	105.1	5.88 (1H, brs)	110.7	6.63 (1H, s)	92.9
5	1.56 (1H, m)	79.6	-	86.9	-	79.2
6	1.88 (1H, m)	46.8	1.95 (1H, br)	46.3	1.85 (1H, m)	46.4
7	1.35, 1.90 (each 1H, m)	24.3	1.45, 2.03 (each 1H, m)	26.4	1.10, 1.92 (each 1H, m)	24.2
8	1.90 (2H, m)	35.2	1.96 (2H, m)	35.3	1.04, 1.95 (each 1H, m)	34.4
9	1.84 (1H, m)	31.3	1.98 (1H, m)	31.0	1.68 (1H, m)	30.7
10	3.06 (1H, qd, J = 9.2, 7.7 Hz)	34.7	2.68 (1H, m)	40.8	3.15 (1H, m)	34.8
11	-	172.0	-	179.3	-	171.4
12	1.13 (3H, d, 8.0 Hz)	12.5	1.51 (3H, d)	16.2	1.19 (3H, d)	12.3

13	0.94 (3H, d, 8.0 Hz)	20.5	1.12 (3H, d)	20.8	0.97 (d)	20.2
OCH_3	3.52 (3H, s)	55.1				
$COCH_3$					2.15 (3H,s)	21.0

[1] P. Pandey, S. Singh, N. Tewari, K.V.N.S. Srinivas, A. Shukla, N. Gupta, P.G. Vasudev, F. Khan, A. Pal, R.S. Bhakuni, S. Tandon, J.K. Kumar, S. Banerjee, Hairy root mediated functional derivatization of artemisinin and their bioactivity analysis, Journal of Molecular Catalysis B: Enzymatic, 113 (2015) 95-103.

[2] Y. Zhan, H. Liu, Y. Wu, P. Wei, Z. Chen, J.S. Williamson, Biotransformation of artemisinin by Aspergillus niger, Appl Microbiol Biotechnol, 99 (2015) 3443-3446.
